# CYP19A1 (aromatase) dominates female gonadal differentiation in chicken (*Gallus gallus*) embryos sexual differentiation

**DOI:** 10.1042/BSR20201576

**Published:** 2020-10-13

**Authors:** Kai Jin, Qisheng Zuo, Jiuzhou Song, Yani Zhang, Guohong Chen, Bichun Li

**Affiliations:** 1 Key Laboratory of Animal Breeding Reproduction and Molecular Design for Jiangsu Province, College of Animal Science and Technology, Yangzhou University, Yangzhou, Jiangsu 225009, China; 2 Institutes of Agricultural Science and Technology Development, Yangzhou University, Yangzhou 225009, China; 3 Joint International Research Laboratory of Agriculture and Agri-Product Safty of Ministry of Education of China, Yangzhou University, Yangzhou 225009, China; 4 Animal and Avian Sciences, University of Maryland, College Park, MD 20741, U.S.A.

**Keywords:** chicken embryonic, Cyp19a1, gonadal differentiation

## Abstract

Cytochrome P450 Family 19 SubFamily A member 1 (*CYP19A1*) gene encodes an aromatase which regulates the sexual differentiation in vertebrates by initiating and maintaining 17β-Estradiol (E_2_) synthesis. Here, we described the spatiotemporal expression pattern of CYP19A1 and its functional role in the embryonic gonad development in amphoteric chickens (*Gallus gallus*). Results showed that CYP19A1 exhibited a sexually dimorphic expression pattern in female gonads early at embryonic day 5.5 (HH 28) and robustly expressed within the cytoplasm in ovarian medullas. Most importantly, we induced the gonadal sex reversal by ectopically delivering the aromatase inhibitor (AI) or estradiol (E_2_) into chicken embryos. To further explore the role of CYP19A1 in chicken embryonic sexual differentiation, we successfully developed an effective method to deliver lentiviral particles with CYP19A1 manipulation into chicken embryos via embryonic intravascular injection. The analysis of interference and overexpression of CYP19A1 provided solid evidences that CYP19A1 is both necessary and sufficient to initiate sex differentiation toward female in chicken embryos. Collectively, this work demonstrates that *CYP19A1* is a crucial sex differentiation gene in the embryonic development, which provides a foundation for understanding the mechanism of sex determination and differentiation in chickens.

## Introduction

In mammals, the well-developed system for single-sex reproduction is based on the known sex-determination system [1,2]. In the dairy industry, most female cows are artificially inseminated with sorted semen to increase the number of female calves aiming to increase dairy milk production by creating an optimal ratio of male and female calves [3,4]. As egg-laying chickens need the female offspring while broiler chickens need males, the specific sex selection helps to reduce the cost and promote the poultry industry development [5]. However, there is still a big challenge to establish an effective sex-selection technology in birds due to the indistinct sex differentiation, causing more than half of poussins eliminated [6]. Therefore, it is essential to identify and characterize the function of critical sex differentiation genes for improving the single-sex reproduction.

Cytochrome P450 Family 19 SubFamily A member 1 (CYP19A1), a vital enzyme for estrogen (17β-Estradiol, E_2_) synthesis in most vertebrates, modulates steroid hormones involved in the sex differentiation among amphibians, fishes, reptiles, birds and mammals [7–10]. In chickens, the manipulation of estrogen levels has been shown to induce the sex reversal [11,12]. Inhibition of estrogens by aromatase inhibitor (AI) may induce a permanent female-male sex reversal which was characterized by the formation of bilateral testis with the spermatogenesis ability and an external male phenotype [13]. In reverse, overexpression of CYP19A1 at the early growth stage of embryos may induce a male-female sex reversal which was characterized by an enlargement of the left gonad and the development of ovarian structure [14]. Moreover, estradiol plays a key role in the development and function of the oviduct by mobilizing calcium for eggshell formation and mediating secondary sexual characteristics [15]. Additionally, the exposure to exogenous estrogen induces feminization in genetically male embryos, but this effect is not permanent [16]. Taken together, aromatase and its product, estradiol, are critically important players involved in the sexual differentiation in chicken embryonic development.

Even though CYP19A1 represents a hallmark of the sexual differentiation in chickens, the function of CYP19A1 in embryonic development has not been systematically reported. In the present study, we described the expression pattern of CYP19A1 and its functional role in chicken embryonic gonads development by mediating the estriol level via AI treatment and manipulating CYP19A1 using lentivirus-mediated RNAi and overexpressing systems. These data indicate that the CYP19A1 is a crucial sex differentiation gene in embryonic development providing insights in better understanding the mechanism of sex determination in chickens.

## Materials and methods

### Materials and animals

All chicken eggs were collected from Rugao Yellow Chicken (Poultry Institute, Chinese Academy of Agricultural Sciences, China). Eggs were incubated at 37°C and 75% relative humidity for 4.5 days (HH 25) and 18 days (HH 44), respectively.

All experiments involving animals in the article were carried out at Chinese Academy of Agricultural Sciences and incubated at the Laboratory of Yangzhou University. Other experiments were performed in the Laboratory of Yangzhou University. Chicken embryos at 4.5 days (HH 25) and 18 days (HH 44) were isolated from incubated eggs. In brief, embryos were exposed by knocking and opening the blunt of eggshell, taken out and placed into the Petri dishes containing PBS. Then, genital ridges or gonads were obtained by peeling off the embryonic membrane and tearing the skin of ventral side using forceps.

### Genetic sex of chicken embryos by PCR

PCR amplification was performed using Mighty Amp DNA Polymerase 2.0 (Takara, Dalian, China, R071A) and extracted cell or tissue samples were used as templates. Primers were designed based on the genomic sequence of chicken *CHD1* gene on sex chromosomes (CHD1-Z, chrZ:51359549-51400046/CHD1-W, chrW: 4989932- 5105612, galGal6a, UCSC). The lengths of our amplified products using our primers for *CHD1* are 580 bp (chrZ: 51387236-51387815) on Z chromosome and 434 bp (chrW: 5019696-5020129) on W chromosome. The sequences of CHD-Forward/Reward primers are as following:

CHD-F: CTGCGAGAACGTGGCAACAGAGT;
CHD-R: ATTGAAATGATCCAGTGCTTG.

In a standard procedure, PCR was performed in a reaction system of 20 μl consisting of 2 μl sample, 10 μl of 2× Mighty Amp Buffer (TAKARA, Beijing, China, DR070), 0.4 μl Mighty Amp DNA Polymerase 2.0 (TAKARA, Beijing, China, DR070), 1 μl CHD-F primer (1 μM) and 1 μl CHD-R primer (1 μM). The condition of PCR was standardized at 98°C for 2 min followed by 30 reaction cycles at 98°C for 10 s, 60°C for 15 s and 68°C for 40 s. PCR products were separated by agarose gel electrophoresis (2% in TBE) and visualized under UV light (Bio-Rad ChemiDoc™ Imaging System, Hercules, U.S.A.) after the Ethidium Bromide staining. Molecular size marker (DL5,000 DNA Marker; TAKARA, Dalian, China, 3428) was used to match the length of PCR products. The genetic sex of chicken embryos was identified by the size of bands: male (ZZ) with one band at 580 bp and female (ZW) with two bands at 580 and 423 bp.

### Sex hormone concentration measurement by ELISA

The concentration of sex hormone was measured using 17 β Estradiol ELISA Kit (Abcam, Cambridge, U.K., ab108667). Allantois fluid or blood samples were collected and concentrations of those samples were measured according to manufacturer’s instructions. Two hundred microliters of 17β estradiol–HRP conjugate was added to each well containing 25 μl standards, and control or experimental samples followed by the incubation for 2 h at 37°C. TMB substrate solution was added to each well after washing for three times using 300 μl diluted washing solution followed by a 30-min incubation at room temperature in the dark. One hundred microliter stop solution was added into each well in the same order and at the same rate as for the TMB substrate solution followed by the gentle shaking for 5 min. Absorbance at 450 nm was measured within 30 min after the addition of stop solution.

### AI and E_2_ treatment

Prior to the injection, AI (Letrozole, Solarbio Life Science, Beijing, China, IL0060) and E_2_ (17β-Estradiol, Solarbio Life Science, Beijing, China, IE0210) were dissolved in dimethyl sulfoxide (DMSO, Solarbio Life Science, Beijing, China, D8370) and diluted in 0.1 ml DMEM (Gibco, Grand Island, U.S.A., 11965) with various doses (0.000, 0.025, 0.050, 0.075 and 0.100 mg).

For chicken embryo injection, fertilized and freshly laid eggs were incubated for 2.5 days (HH 17) and then the eggshell was gently wiped off using sanitized cotton with 75% ethanol. A small window (<0.5 cm diameter, it is good to keep the window as small as possible) was made by using forceps sharpened at the broader edge (blunt end). Prepared AI or E2 solutions were injected into eggs under the air sac with 1-ml syringes. Holes were sealed with hot-melted paraffin or scotch tape followed by the egg incubation for 4.5 days (HH 25) or 18 days (HH 44).

### Construction and delivery of interference and overexpression vectors

According to the coding region of chicken *CYP19A1* gene (NCBI gene ID: 395783, accession: NM_001364699), three specific shRNAs targeting CYP19A1 were designed by Shanghai Gima Gene Corporation and then ligated into the pGMLV-SC5 vector carrying a gene encoding GFP. Similarly, CYP19A1-overexpressing plasmid was generated by inserting the coding sequence (CDS) of CYP19A1 into the pLV-OE vector containing the CDS of GFP. High-titer lentiviral particles (10^8^ Tu/ml) carrying shRNAs targeting CYP19A1 or CYP19A1-overexpressing plasmid were generated in Lenti-X 293T packaging cells with packaging vectors (pLP1 and pLP2) and envelope vector (pLP/VSVG).

After the incubation for 2.5 days (HH 17), a small window (<1 cm diameter) was made using forceps sharpened at the broader edge (blunt end) to expose chicken embryos. Five microliters of lentiviral solution was injected into the upper portion of the dorsal aorta of the recipient embryo using mouth pipette; 400–600 μl of penicillin–streptomycin solution was dropped on the top of embryos which were further incubated until 4.5 days (HH 25) and 18 days (HH 44) after sealing the window using scotch tapes. The efficiency of virus infection was determined by measuring the intensity of GFP using a stereo-fluorescence microscope (MVX10, Olympus, Tokyo, Japan). Gonads or gonad–middle–renal complexes were isolated from embryos at different stages for RNA extraction and histomorphological analysis.

### Quantitative real time polymerase chain reaction

Total RNA from collected tissues was extracted using TRIzol reagent (Invitrogen, Carlsbad, U.S.A., 15596-026). cDNA was synthesized from 1 μg total RNA using RevertAid First Strand cDNA Synthesis Kit (MBI/Fermentas, Lithuania, K1621) according to the manufacturer’s instructions. Real-time PCR was performed using SuperReal PreMix Plus (SYBR Green) (TIANGEN, Beijing, China, FP205). Gene expression levels were presented as relative values. All the experiments were performed in triplicate. The sequences of quantitative real time polymerase chain reaction (qRT-PCR) primers were listed in Supplementary Table S1.

### Morphology and HE

The removed embryonic gonads were stored in 4% paraformaldehyde, fixed for 24 h and then transferred to 50% ethanol. The tissue shape was corrected and photographed under a stereo-microscope (MVX10, Olympus, Tokyo, Japan). After the ethanol concentration was dehydrated from low to high gradient, xylene was transparent, paraffin was embedded and conventional paraffin section was cut with a thickness of 5–6 μm. Sections were dewaxed with xylene and rehydrated using ethanol with gradient concentration from high to low followed by Hematoxylin–Eosin staining, dehydration and mounting slides. Sections were visualized using the microscope (Eclipse 80i, Nikon, Tokyo, Japan).

### Immunofluorescence staining

After dewaxing with xylene, slides were infiltrated through ethanol from high to low concentrations and then placed into a 0.01 mol/l sodium citrate solution. After repairing the antigen at 95°C for 20 min, slides were cooled to room temperature and incubated with blocking buffer (10% normal donkey serum, 3% bovine serum albumin, 0.3% Triton X-100) at room temperature for 1 h. Then slides were stained with anti-SOX9 antibody (1:500, Abcam, Cambridge, U.K., ab3697) or anti-aromatase antibody (1:250, Abcam, Cambridge, U.K., ab139492) overnight at 4°C. After three washes using phosphate Triton X-100 buffer (PBST) for 10 min each time, slides were stained with sheep anti-rabbit IgG-488 (1:250, Invitrogen, Carlsbad, U.S.A., 11203D) or goat anti-mouse IgG H&L (DyLight® 594) (1:1000, Abcam, Cambridge, U.K., ab96873) for 1 h at room temperature in the dark followed by three PBST washes. DAPI (286 nmol/l, Sigma, St. Louis, U.S.A., D9542) was used to stain the nucleus at room temperature for 5 min in the dark. Following three PBST washes, slides were mounted with anti-fluorescence quenching solution and visualized using a confocal fluorescence microscope (A1 Plus, Nikon, Tokyo, Japan).

## Results

### The sexually dimorphic expression pattern of *CYP19A1* gene in chicken (*Gallus gallus*)

**Figure 1:**
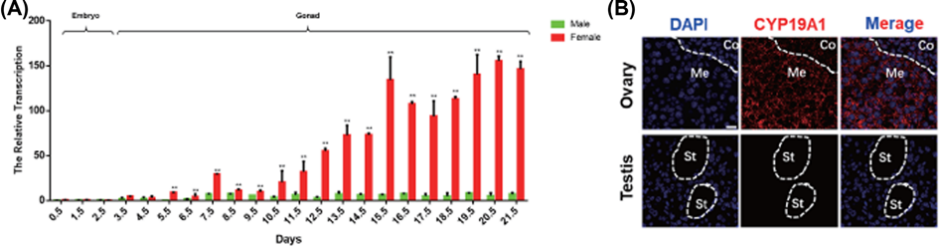
The sexually dimorphic expression pattern of *CYP19A1* gene in chicken (*Gallus gallus*) (**A**) The mRNA expression of CYP19A1 in embryos (days 0–2.5, HH 0–17) and gonads of different stages (days 2.5–21.5, HH 17–48), determined by qRT-PCR analysis; β-actin was used as a reference gene. (**B**) Immunofluorescence detection of CYP19A1 protein in the gonad at day 18.5 in female and male. CYP19A1 protein was localized in the cytoplasm of the medulla of the female gonad (ovary, day 18.5, HH 44). Co, cortex; Me, medulla; St, seminiferous tubule. Scale bar: 10 μm (data were shown as mean ± SEM and Student’s *t* test was utilized for statistical analysis.**P<0.01).

In order to determine the expression patterns of *CYP19A1* (aromatase) in chicken (*Gallus gallus*) embryonic development, the spatiotemporal expression of CYP19A1 was evaluated by qPCR. The results showed that the CYP19A1 transcript was significantly highly expressed in female gonads throughout the period of sex determination to born, from as early as day 5.5 (HH 28) to the day 21.5 (born), but barely expressed in male gonad (Figure 1A). Moreover, CYP19A1 was localized in the medulla cells in the early ovary, by contrast, the signal was not detectable in early testis (Figure 1B). These data reveal that the sexually dimorphic expression pattern of CYP19A1 and differed in chicken early ovary, imply CYP19A1 involved in ovary development in chicken (*Gallus gallus*), as in other species [17,18].

### The effect of AI and E_2_ on embryonic gonad development

**Figure 2:**
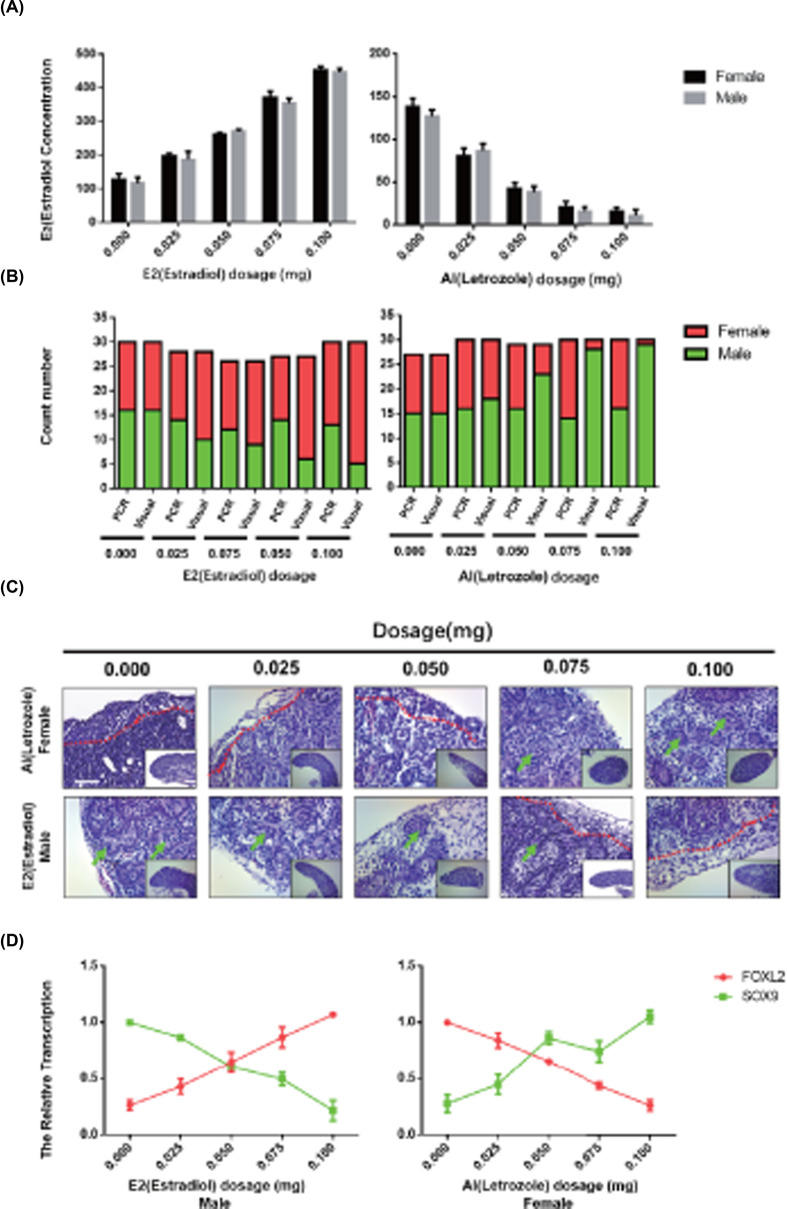
The effect of AI and Estradiol (E_2_) on embryonic gonad development (**A**) The concentration of Estradiol in different treatments. (**B**) The ratio of PCR and visual results in different treatment groups. (**C**) The histology of E18.5 gonads in different treatments (the red dot line separates the cortex and medulla; the green arrow points the spermatic cord). Scale bar: 50 μm. (**D**) The Sox9 and Foxl2 expression in different treatments (data were shown as mean ± SEM and Student’s *t* test was utilized for statistical analysis. *P<0.05, **P<0.01).

Previous studies demonstrated that AI led to inhibit the CYP19A1 expression and decrease in estradiol [10]. We examined the genetic sex by PCR (Supplementary Figure S1A) and used different concentrations of AI (Aromatase Inhibitor, Letozole) and estradiol (E_2_, estradiol) to treat day 2.5 (HH 17) chicken embryos to sync the level of the estradiol (Figure 2A). To investigate the sex of chicken embryo-sex transformation after the treatment, we developed a method by morphology based on the length of the gonads on both sides of the normally developing male (ZZ) chicken embryos which was similar and the female (ZW) left gonads that were larger and the right gonads smaller. We determined that the gonad area ratio (left/right) was 1.21 + 0.23 for male, and 2.75 + 0.44 for female (Supplementary Figure S1B,C). Compared with the PCR and morphological results, the gradually elevated E_2_ induced the increased proportion of female and AI was the opposite (Figure 2B and Supplementary Figure S1D). The masculinization in female gonads after AI treatment, the cortex thinning (red dot line) and spermatic cord (green arrow) appeared in the female left gonad. In contrast, feminization in male gonads after E_2_ showed the female structure (spermatic cord disappeared and cortex thickened) (Figure 2C). With the reversal of the sex of the gonads, the expression of the sex marker genes in the gonads also reversed. Specifically, the expression of Sox9 in AI-treated ZW gonads gradually increased and the expression of Foxl2 decreased. The E2 treatment in ZZ gradually increased the Sox9 and decreased the Foxl2 (Figure 2D). These observations indicate that CYP19A1 is a vital factor for the induction of embryonic gonad development in chicken.

### Establishment of an efficient lentivirus-mediated gene modulating method in chicken

**Figure 3:**
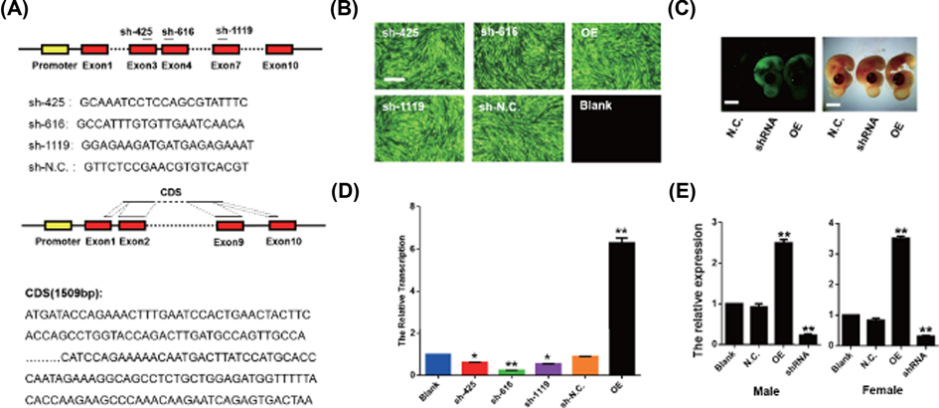
Establishment of an efficient lentivirus-mediated gene modulating method in chicken (*Gallus gallus*) (**A**) Schematic diagram of CYP19A1 target sites of RNAi and CDS. (**B**) The efficiency of virus infection in DF-1 cells of each experimental group and control group. (**C**) CYP19A1 mRNA relative expression in DF-1 cells of each experimental group and control group. (**D**) The expression of EGFP by fluorescent microscopy at day 4.5 (HH 25). (**E**) The expression of CYP19A1 in gonad different groups (data were shown as mean ± SEM and Student’s *t* test were utilized for statistical analysis. *P<0.05, **P<0.01).

To solve the problem of the lack of available genetic manipulation techniques in chicken and verify the effect, we established an efficient gene-modulating method for functional analysis *in ovo*. We developed lentiviral vectors carrying CYP19A1-specific shRNAs with a GFP reporter gene to knockdown and encode the CDSs for overexpression of the endogenous CYP19A1 transcripts and verify the vector activity in DF-1 cells (Figure 3A–C). To test the activity of this virus system *in ovo*, the expression of EGFP was detected in embryos at day 4.5 (HH 25) of development, indicating that the virus is efficient (Figure 3D). Quantitative results showed that the expression of CYP19A1 decreased significantly after treatment with CYP19A1 interference in female and male chicken gonads, while overexpression induces the increase in CYP19A1 (Figure 3E). These results indicate that we successfully developed an effective gene up- and down-modulating method in chicken that works both *in vitro* and *in ovo*.

### Feminization of male embryo gonads following *CYP19A1* knockdown and masculinization of female *CYP19A1* overexpressing *in ovo*

**Figure 4:**
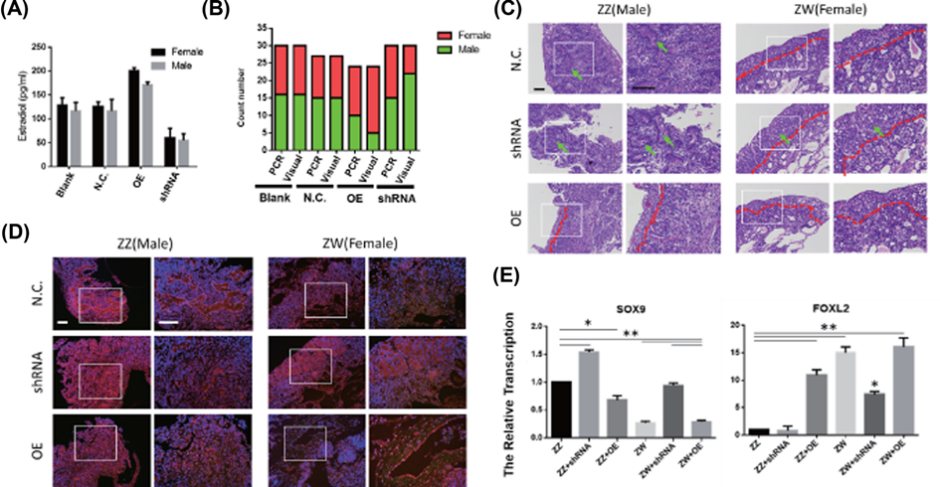
Feminization of male embryo gonads following CYP19A1 knockdown and masculinization of female overexpressing CYP19A1 *in ovo* (**A**) The concentration of estradiol in different groups. (**B**) The ratio of PCR and visual in different treatment groups. (**C**) The histology of E18.5 gonads in different treatments (the red dot line separates the cortex and medulla; the green arrow point the spermatic cord). Scale bar: 50 μm. (**D**) Immunofluorescence detection of SOX9 (green) in the gonads at different groups. Scale bar: 50 μm. (**E**) The expression of Sox9 and Foxl2 at different groups (data were shown as mean ± SEM and Student’s *t* test were utilized for statistical analysis. *P<0.05, **P<0.01).

To verify the function of CYP19A1 in the process of gonad differentiation in chicken embryos, we infected the scrambled virus overexpression or interference to the chicken embryo by vessel injection method and compared phenotype and marker gene expression by gonadal histology, immunofluorescence and qRT-PCR at day 18.5 (HH 44). We examined the effects of CYP19A1 overexpression and interference on embryonic estradiol. qRT-PCR results showed that the overexpression of CYP19A1 causes up-regulation of estradiol in both females and males, and interference will reduce the concentration of estradiol (Figure 4A). It is demonstrated that the CYP19A1 regulates estradiol synthesis during embryonic development. In addition, the sex phenotype is also transformed with the overexpression and interference of CYP19A1, more female embryos were observed in CYP19A1 overexpression and more male in CYP19A1 interference (Figure 4B). Compared with the normal male gonads (ZZ), the left swollen gonad appeared after overexpressing CYP19A1 in male. The section results further indicate that early spermatic cord disappeared and cortex thickened. Reversed after interfering with CYP19A1 in female chicken embryos, no significant difference in the size of the two gonads was similar to the morphology of the male gonads. The section results showed that the female chicken embryonic gonads exhibited cortex thinning (red dot line) and spermatic cord (green arrow) appeared in the female left gonad after interfering with CYP19A1 (Figure 4C and Supplementary Figure S2). The results of immunofluorescence showed that overexpression of CYP19A1 inhibits the expression of Sox9, while interference could promote the expression of Sox9 in the gonads (Figure 4D). Moreover, the results of qRT-PCR also showed that Sox9 was highly expressed in control males and female with CYP19A1 interfering and the Foxl2 was highly expressed in control female and male with CYP19A1 overexpression (Figure 4E). These results indicate that CYP19A1 plays a critical role in chicken sexual differentiation, overexpression of CYP19A1 promote the differentiation of female gonads, while interference promotes the differentiation of male gonads.

## Discussion

**Figure 5:**
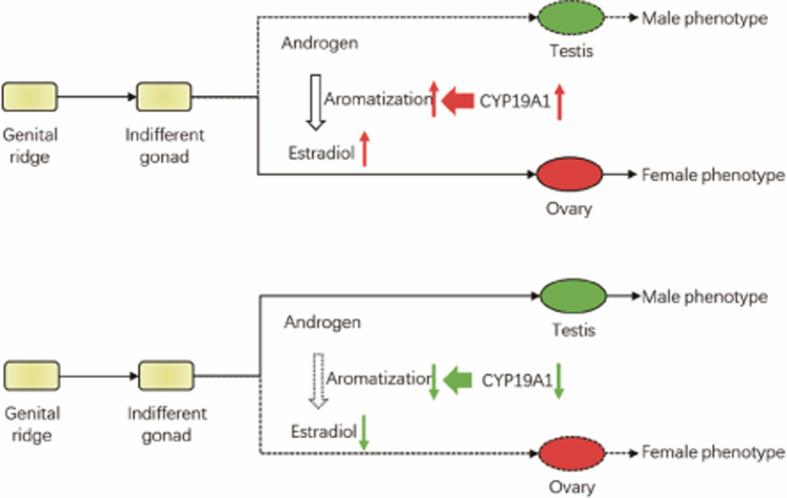
The schematic diagram of CYP19A1 function in chicken sexual differentiation

Even though the bird sexual differentiation has been studied for several decades, the molecular mechanism has remained elusive. Here, we demonstrate that CYP19A1 acts as a female sex differentiation gene in chicken and showed that CYP19A1 has sexually dimorphic expression pattern and high expression in the medulla of female embryonic gonad (day 18.5, HH 44). Most importantly, we provided solid functional evidence that CYP19A1 is both necessary and sufficient to initiate female development in chicken (*Gallus gallus*) embryonic via a novel *in ovo* viral transduction system and treatment of AI/Estradiol. This is the first integrity and systematic research of the function characterization of CYP19A1 (Aromatase) in sexual differentiation in chicken (*Gallus gallus*) embryonic development (Figure 5).

It has been demonstrated that the sexual differentiation in birds is quite different from that in mammals, which have symmetrical ovarian development. However, birds lose the right ovary and oviduct in the sexual development [19]. In chicken embryos, male and female gonads are morphologically indistinguishable in appearance in day 5.5 (HH 28) [12]. We found that CYP19A1 also exhibited early female-specific embryonic expression before the onset of gonadal sex differentiation in the chicken embryos and express the medulla cells of ovary. These results consistent with previous findings demonstrate that the CYP19A1 is female-specific and expresses in early gonads from the time of gonadal sex differentiation in chicken [20–23]. These features of CYP19A1 expression suggest that it is important for both primary sex differentiation and subsequent gonadal differentiation in chicken.

Gonadal steroid hormones are crucial for sexual differentiation of endocrine components of reproduction [24]. In birds, estradiol appears to be critical in the sexual differentiation of females [25]. The current study shows that the manipulation of estradiol levels in the early chicken embryo induces the female-to-male or male-to-female sex reversal [26]. The exogenous estradiol and its synthetase aromatase can override the genetic effect if applied during the sex differentiation [27]. In this study, we developed a method to identify the phenotype of embryonic sex and demonstrate the effect of AI and Estradiol (E_2_) on embryonic gonad development. Moreover, in lizard and Brazilian turtle embryos, the estradiol and AIs induced sex reversion [28]. In *Xenopus laevis*, the exogenous AIs induce female gonads to develop male traits [29,30]. However, in mammals, injection of estradiol or estradiol inhibitors does not promote permanent sexual reversal and once the injection of hormones or inhibitors is stopped, the sex phenotype will still return to its original [31,32]. This suggests that the effects of sex hormones may not be as important in Genetic-Sex-Determined (GSD) animals as in Environment-Sex-Determined (ESD) animals. The dose-dependent AIs that induce the sex phenotype reversal in adult chicken also have been identified. Our results showed that although the chicken sex determination is GSD but the hormones conduce permanent sex reserved. The dose-dependency on hormones in chicken confirm that the chicken sex differentiation and maintenance were involved by hormones and different from mammals, but similar to reptiles.

We developed a viral vector for overexpression and interference *in ovo*, which upon injection into vascellum of day 2.5 (HH 17) embryos allow for embryo-wide infection and transgene expression. The immunofluorescence performed the vector infection and qRT-PCR showed increased or decreased levels of CYP19A1 expression, indicating the validity of this viral system. The data presented here clearly showed that CYP19A1 is a strong master gene in chicken female gonadal differentiation, and independently initiate male gonadal differentiation to female, while female gonadal differentiation into male with the expression suppressed. Moreover, CYP19A1 affects the expression of sex downstream gene *Sox9* and *Foxl2*, suggesting that CYP19A1 is a key factor that direction differentiation of embryonic sex development in chicken. In *Xenopus laevis*, knockout of CYP19A1 induce the whole-male offspring, which indicate the CYP19A1 is a key regulator of zebrafish female sexual gonads differentiation [33]. In the *Pelodiscus sinensis*, the overexpression and interference of CYP19A1 lead to the sex reversal [34]. The overexpression of CYP19A1 induce the ovarian development in males [14]. These data suggest that the CYP19A1 as a regulator may be highly conserved in the early ovarian differentiation in non-mammalian vertebrates.

## Conclusion

Although the importance of CYP19A1 in maintaining female gonadal development has been well documented, the present study systematically performed that CYP19A1 overexpression or interference can affect gonad phenotype in chicken. This work therefore not only builds the foundation for related research on chicken sex differentiation and mechanism, but also establishes an effective sex reversal model (interference or overexpression of hormones and CYP19A1), which provides a new opportunity for poultry production. It is a potential to obtain all-male offspring in broiler chicken production by mating between CYP19A1 interference individual male with males.

## Supplementary Material

Supplementary Figures S1-S2 and Table S1

## Abbreviations

AI: Aromatase Inhibitor
CDS: Coding Sequence
CYP19A1: Cytochrome P450 Family 19 Subfamily A Member 1
DAPI: 4′,6-Diamidino-2-Phenylindole, Dihydrochloride
EGFP: Enhanced Green Fluorescent Protein
E2: 17β-Estradiol
GSD: Genetic-Sex-Determined
HE: Hematoxylin-Eosin staining
HH: Hamburger and Humilton tage
PBST: Phosphate Buffer Saline with Triton X-100 Buffer
qRT-PCR: Quantitative Real time Polymerase Chain Reaction
TMB: 3,3′,5,5′-Tetramethylbenzidine
