# Supplementary material for: CYP19A1 (aromatase) dominates female gonadal differentiation in chicken (Gallus gallus) embryos sexual differentiation
Source: Biosci Rep. 2020 Oct 13;40(10):BSR20201576. doi: 10.1042/BSR20201576 (PMC7560524; doi:10.1042/BSR20201576)
Supplement: Supplementary Figures S1-S2 and Table S1 [file BSR-2020-1576_supp.pdf]

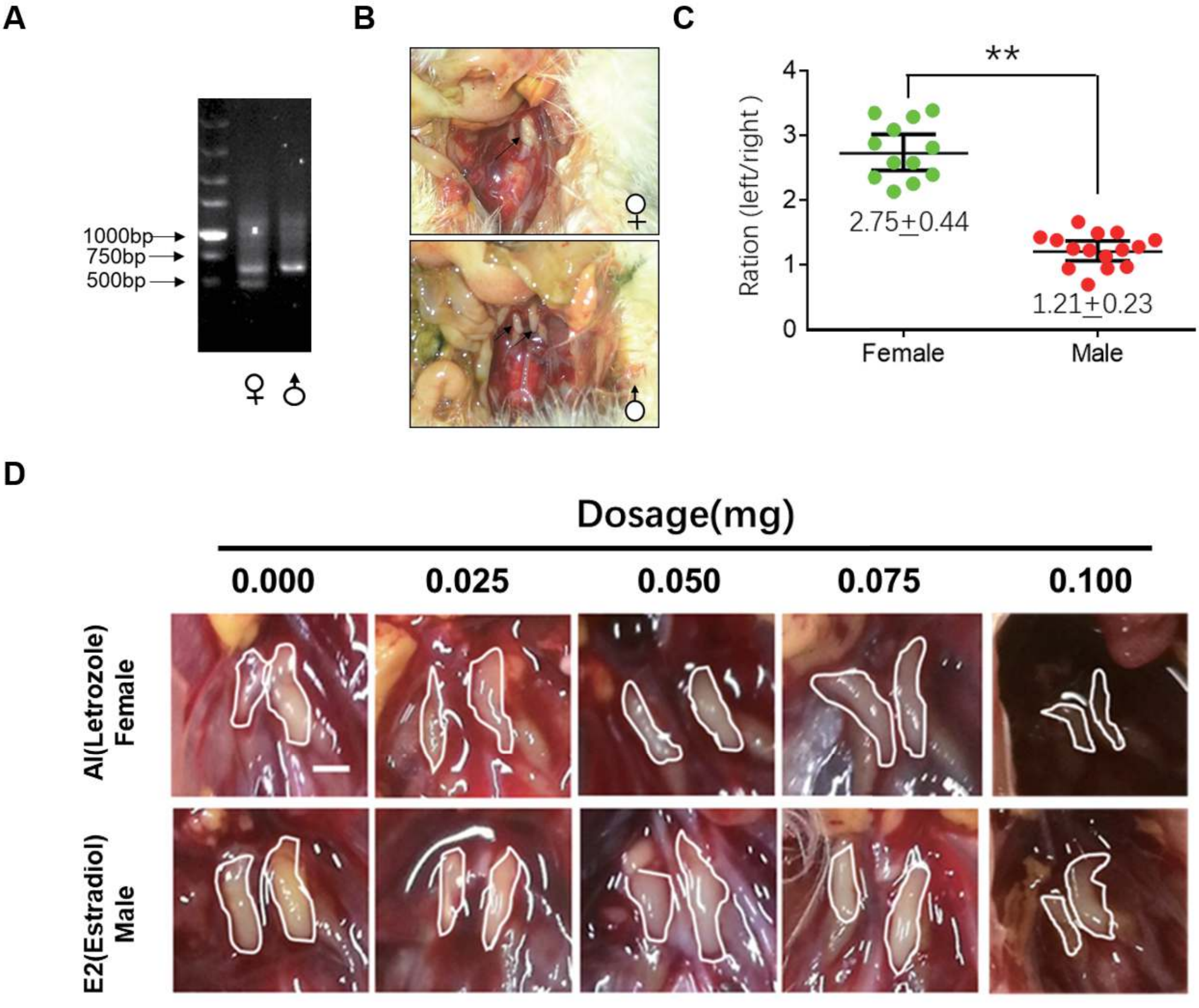

A
1000bp
750bp
500bp
♀
♂
B
♀
♂
C
**
Ration (left/right)
4
3
2
1
0
2.75±0.44
1.21±0.23
Female
Male
D
Dosage(mg)
0.000
0.025
0.050
0.075
0.100
AI(Letrozole) Female
E2(Estradiol) Male

Supplementary Figure 1. The sex identify protocol of embryonic. (A) The CHD1amplification of gDNA, one band is male (ZZ) and two bands is female (ZW). **(B)**The morphologic view of gonads in different sex at day18.5(HH 44). **(C)**The analysis of the ration(left/right)in different sex at day18.5(HH 44).**(D)**The morphologic view of gonads in different treatments at day18.5(HH 44).

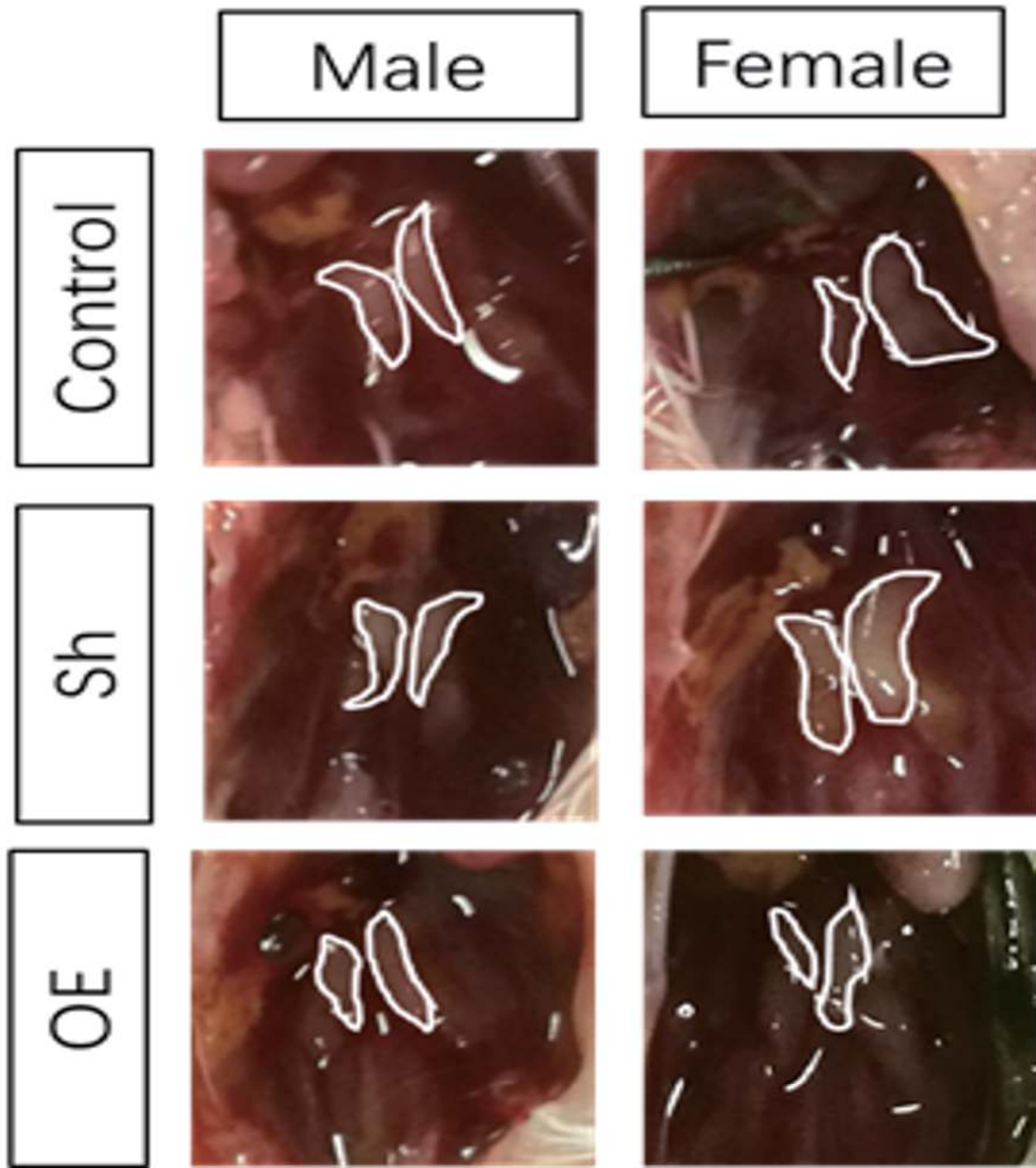
Male
Female
Control
Sh
OE

Supplementary Figure 2. The morphologic view of gonads in different treatments at day18.5(HH 44).

Supplementary Table1. Primes for qRT-PCR

Gene		Primer Sequence（5'-3'）
CYP19A1	F:	TGTTCCATCACGCTATTT
	R:	GATTCTTGTTTGGGCTTC
SOX9	F:	AAGGAGAGCGACGAGGACAAA
	R:	GGTCCAGTCGTAGCCCTTGA
FOXL2	F:	CCTCAACGAGTGCTTCATCA
	R:	ACATCTGGCAAGAGGCGTAG
β-actin	F:	CAGCCATCTTTCTTGGGTAT
	R:	CTGTGATCTCCTTCTGCATCC
